# Important mitochondrial proteins in human omental adipose tissue show reduced expression in obesity

**DOI:** 10.1016/j.dib.2015.04.007

**Published:** 2015-04-23

**Authors:** Peter W. Lindinger, Martine Christe, Alex N. Eberle, Beatrice Kern, Ralph Peterli, Thomas Peters, Kamburapola J.I. Jayawardene, Ian M. Fearnley, John E. Walker

**Affiliations:** a Laboratory of Endocrinology, Department of Biomedicine, University Hospital and University Children’s Hospital, University of Basel, Basel CH-4031, Switzerland; b Department of Surgery, St. Claraspital, Basel CH-4059, Switzerland; c Interdisciplinary Center of Nutritional and Metabolic Diseases, St. Claraspital, Kleinriehenstrasse 30, Basel CH-4058, Switzerland; d MRC Mitochondrial Biology Unit, Welcome Trust/MRC Building, Hills Road, Cambridge CB2 0XY, UK; e Collegium Helveticum, ETH Zurich, Zurich CH-8092, Switzerland

## Abstract

Obesity is associated with impaired mitochondrial function. This study compares mitochondrial protein expression in omental fat in obese and non-obese humans. Omental adipose tissue was obtained by surgical biopsy, adipocytes were purified and mitochondria isolated. Using anion-exchange chromatography, SDS-PAGE and mass-spectrometry, 128 proteins with potentially different abundances in patient groups were identified, 62 of the 128 proteins are mainly localized in the mitochondria. Further quantification of 12 of these 62 proteins by immune dot blot analysis revealed four proteins citrate synthase, HADHA, LETM1 and mitofilin being inversely associated with BMI, and mitofilin being inversely correlated with gender.

## 1 Specifications table

**Table:** 

Subject area	Biology
More specific subject area	Mitochondrial function in humans
Type of data	Table
How data was acquired	Proteome analysis by anion-exchange chromatography, SDS-PAGE and mass-spectrometry
Data format	Analyzed
Experimental factors	Surgical biopsy, isolation of omental adipocytes , mitochondrial purification
Experimental features	Purification of adipocytes by collagenase digestion, filtration and centrifugation. Isolation of mitochondria by lysis with ultrasound, concentration by differential centrifugation. Protein fractionation and preliminary quantification by FPLC, SDS-Page. Protein identification and preliminary quantification by MALTI-TOF mass spectrometry. Final quantification by immunoassay
Data source location	Basel, Switzerland
Data accessibility	Data is available with this article

## 2 Value of the data

• First proteome analysis on human omental adipose tissue.
• Significantly reduced expression of four proteins may explain reduced mitochondrial function of omental fat in obese humans.
• 50 additional mitochondrial proteins, which showed differential expression by SDS-Page, require further quantification.
• The differentially expressed proteins should also be studied in obesity related diseases.
• The findings may stimulate research on the pathophysiology of obesity and may help to develop targeted therapies.

## 3 Data, experimental design, materials and methods

The data shown here are two lists of cellular and mitochondrial proteins found by FPLC, SDS-Page and mass spectrometry, that are differentially expressed in omental adipose tissue of obese and non-obese humans. Supplementary Table 1 contains a list of all 126 proteins found and includes also proteins of non-mitochondrial origin. Supplementary Table 2 contains all 62 mitochondrial proteins with potentially different abundance [1].

### 3.1 Patients and tissue samples

A total of 76 obese and non-obese non-diabetic patients undergoing surgery at the St. Claraspital Basel, Switzerland, were included in this study. The number of 76 biopsies allowed sorting of the samples according to BMI, gender and age of patients. Omental adipose tissue biopsies of at least the size of a chicken egg were collected during bariatric or conventional visceral surgery and immediately processed by purifying the adipocytes and isolating their mitochondria.

### 3.2 Purification of adipocytes

Minced human adipose tissue was resuspended in modified Krebs Ringer buffer (MKRB) (5 mM d-glucose, 2% BSA, 100 mM Hepes, 100 mM KCl, 123 mM NaCl, 1.3 mM CaCl_2_) containing collagenase type 2 (1 mg/ml) and subjected to digestion of connective tissue on an orbital shaker at 37 °C. After digestion, cells were separated from connective tissue by serial filtrations through a tea strainer equipped with fine nylon mesh. Adipocytes were separated from other cells by mild centrifugation, leaving adipocytes on top of the cell suspension. Isolated adipocytes were washed three times with MKRB, followed by a PBS wash step.

### 3.3 Isolation of mitochondria

Purified adipocytes were resuspended in homogenization buffer (HB) (200 mM mannitol, 50 mM sucrose, 1 mM EDTA, 20 mM Hepes, pH 7.4). Lysis was performed with two soft ultrasonic pulses. After centrifugation at 700*g* for 11 min, the post-nuclear supernatant was collected and further centrifuged at 7000*g* for 19 min. Subsequently, the pelleted mitochondria were resuspended in HB and recentrifuged at 7000*g* for 19 min. Finally, the mitochondria were resuspended in HB and the protein concentration was measured using bicinchoninic acid (BCA) protein assay. The protein yields obtained from mitochondrial preparations were related to the mass of the biopsy. More than 1 mg of enriched mitochondrial protein was obtained from 20 clinical samples and less than 1 mg from the remaining 56 samples. A more rigorous purification of mitochondria from these samples was prevented by limitations in sample availability.

### 3.4 Mitochondrial proteome analysis

Comparative proteome analysis was performed on the subset of 20 patients with a higher protein yield, which were divided into 7 non-obese (BMI<30 kg/m^2^) and 13 obese patients (BMI≥30 kg/m^2^).

#### 3.4.1 Fractionation

An Äkta *Explorer* FPLC system equipped with a 1-ml HiTrap *Q* HP column (GE Health Systems) was used to fractionate the mitochondrial proteome by anion-exchange chromatography applying a salt gradient (0–1 M NaCl) in buffer A (20 mM Tris, 1 mM EDTA, 1 mM DTT, 1 g/l *n*-dodecyl-beta-d-maltoside (DDM), 10% glycerol). Approximately 1 mg of mitochondrial protein was required for each fractionation. First, proteins were solubilized in 10 vol buffer A supplemented with additional DDM (0.5% final concentration). The suspension was kept rotating at 4 °C for 20 min, followed by filtration using Spin-X columns (0.22 μm; Costar). The filtrate was applied to the FPLC system and eluted with increasing concentrations of NaCl from 0–0.23 M, 0.23–0.25 M, to 0.25–1 M in buffer A at a flow rate of 0.5 ml/min; 62 fractions (each of 500 µl) were collected. The robustness and reproducibility of this fractionation method was verified in a series of preliminary experiments (data not shown).

#### 3.4.2 Protein precipitation

Proteins in anion-exchange fractions were concentrated for subsequent gel electrophoresis by precipitation with TCA/DOC/acetone. A 1/100 vol of 2% sodium deoxycholate (DOC) was added, followed by incubation at 4 °C for 30 min and addition of 1/10 vol of 100% trichloroacetic acid (TCA). The solution was vortexed and incubated at 4 °C overnight. Proteins were collected by centrifugation (16,000*g*, 4 °C, 10 min), the supernatant was removed and the protein pellets washed with ice-cold acetone. After recentrifugation (16,000*g*, 4 °C, 10 min), removal of the supernatant and air-drying of the tubes, the pellets were dissolved directly in 2× SDS gel loading buffer [2].

*SDS-PAGE.* Proteins were resolved by SDS-PAGE containing gradients of polyacrylamide (12–22%). Protein bands were detected by staining with Coomassie blue dye [2]. Then gels were scanned and inspected for variations in protein patterns. Protein bands of interest were excised and identified by peptide mass fingerprinting and mass spectrometry.

#### 3.4.3 Mass spectrometry

Selected gel bands were proteolysed with trypsin using an in-gel digestion protocol [3], except that ammonium bicarbonate buffers were replaced with 20 mM Tris HCl, pH 8.0, and no alkylation of cysteine residues was performed. Digests were analysed in a MALDI-TOF-TOF 4800 PLUS mass spectrometer (Applied Biosystems) with α-cyano-4-hydroxy *trans* cinnamic acid as the matrix. Spectra were calibrated using the trypsin autolysis peptides at 2163.057 and 2273.160 Da, and a calcium related matrix ion at 1060.048 Da. Peptide sequence data were obtained using the same instrument, by collision induced dissociation, at a collision energy of 1 kV. The MASCOT algorithm (Matrix Science) was used to identify the proteins by comparison of both peptide mass and peptide sequence data with the National Center for Biotechnology Information (NCBI) sequence database [4].

### 3.5 Quantification of protein levels by immunoassay

#### 3.5.1 Selection of proteins and antibodies

The proteins identified by mass spectrometry from the selected 20 patients were sorted according to known functions in mitochondria and the availability and specificity of antibodies for immune detection in a spot assay. Selected proteins and corresponding antibodies are listed in Supplementary Table 2. Quantification of these proteins was extended by applying the immunoassay to mitochondrial preparations from an additional 56 patient samples. All antibodies were extensively checked for specificity in Western blots prior to immunoassay to ensure reliability using a control sample of heart mitochondria (MitoSciences) and mitochondria purified from adipocytes of human omental adipose tissue. Only antibodies reacting with one specific band at the appropriate molecular weight were included in the study. The secondary Li-Cor IRDye antibodies (926-32211; goat anti-rabbit and 926-32210; goat anti-mouse) were coupled to infrared labels. For citrate synthase, a secondary goat anti-sheep antibody coupled to horseradish peroxidase was applied.

#### 3.5.2 Immunoassay

A series of dilutions of mitochondrial preparations with known protein concentrations were used in this assay. Only measurements within the linear range of the assay were included. Several dilutions of mitochondrial preparations and protein standards were spotted on nitrocellulose membranes. After drying of the proteins, the membranes were blocked by 5% skimmed milk solution in TBS/Tween (0.1%) (TBST). Following three wash steps with TBST, the membranes were incubated at 4 °C overnight with antibodies against selected candidate proteins. After three wash steps with TBST, the appropriate secondary antibody was applied (see above). Following three wash steps using TBST, the infrared signals were determined with an Odyssey imaging instrument (Li-Cor Biosciences). Based on these signals, protein levels were calculated. In the case of chemoluminescence, the developed film was scanned and spots were quantified using Quantity One software from Biorad.
